# Anticancer Drug 2-Methoxyestradiol Protects against Renal Ischemia/Reperfusion Injury by Reducing Inflammatory Cytokines Expression

**DOI:** 10.1155/2014/431524

**Published:** 2014-08-06

**Authors:** Ying-Yin Chen, Ching-Hua Yeh, Edmund Cheung So, Ding-Ping Sun, Li-Yun Wang, Chung-Hsi Hsing

**Affiliations:** ^1^Department of Medical Research, Chi-Mei Medical Center, Tainan 710, Taiwan; ^2^Department of Biotechnology, National Formosa University, Yunlin County 632, Taiwan; ^3^Department of Medicinal Botanicals and Health Applications, Da-Yeh University, Changhua 515, Taiwan; ^4^Department of Anesthesia & Medical Research, China Medical University, An Nan Hospital, Tainan 710, Taiwan; ^5^Department of Surgery, Chi-Mei Medical Center, Tainan 710, Taiwan; ^6^Department of Anaesthesiology, Chi-Mei Medical Center, Tainan 710, Taiwan; ^7^Department of Anesthesiology, Taipei Medical University, Taipei 110, Taiwan

## Abstract

*Background*. Ischemia/reperfusion (I/R) injury is a major cause of acute renal failure and allograft dysfunction in kidney transplantation. ROS/inflammatory cytokines are involved in I/R injury. 2-Methoxyestradiol (2ME2), an endogenous metabolite of estradiol, inhibits inflammatory cytokine expression and is an antiangiogenic and antitumor agent. We investigated the inhibitory effect of 2ME2 on renal I/R injury and possible molecular actions. *Methods*. BALB/c mice were intraperitoneally injected with 2ME2 (10 or 20 mg/kg) or vehicle 12 h before and immediately after renal I/R experiments. The kidney weight, renal function, tubular damages, and apoptotic response were examined 24 h after I/R injury. The expression of mRNA of interleukin-1*β*, tumor necrosis factor- (TNF) *α*, caspase-3, hypoxia inducible factor- (HIF) 1*α*, and proapoptotic Bcl-2/adenovirus E1B 19 kDa interacting protein 3 (BNIP3) in kidney tissue was determined using RT-PCR, while the expression of nuclear factor *κ*B (NF-*κ*B), BCL-2, and BCL-xL, activated caspase-9, and HIF-1*α* was determined using immunoblotting. *In vitro*, we determined the effect of 2ME2 on reactive oxygen species (ROS) production and cell viability in antimycin-A-treated renal mesangial (RMC) and tubular (NRK52E) cells. *Results*. Serum creatinine and blood urea nitrogen were significantly higher in mice with renal I/R injury than in sham control and in I/R+2ME2-treated mice. Survival in I/R+2ME2-treated mice was higher than in I/R mice. Histological examination showed that 2ME2 attenuated tubular damage in I/R mice, which was associated with lower expression TNF-*α*, IL-1*β*, caspase-9, HIF-1*α*, and BNIP3 mRNA in kidney tissue. Western blotting showed that 2ME2 treatment substantially decreased the expression of activated caspase-9, NF-*κ*B, and HIF-1*α* but increased the antiapoptotic proteins BCL-2 and BCL-xL in kidney of I/R injury. *In vitro*, 2MR2 decreased ROS production and increased cell viability in antimycin-A-treated RMC and NRK52E cells. *Conclusions*. 2ME2 reduces renal I/R injury in mice because it inhibits the expression of ROS and proinflammatory cytokines and induces antiapoptotic proteins.

## 1. Introduction

Ischemia/reperfusion (I/R) injury during renal transplantation is a major cause of acute kidney injury (AKI) and is associated with increased morbidity and mortality [1]. Several studies have reported that ischemia followed by reperfusion initiates changes in the renal oxygen balance and leads to further detachment of vascular endothelial cells from the endothelial monolayer to tubular epithelial injury and to inflammation-induced vascular congestion that extends the hypoxic region and reduces the clearance of oxygen-derived radicals in the kidney [2]. I/R injury is more destructive than ischemia alone because it causes apoptosis of endothelial and proximal tubular epithelial cells (TECs) after the reperfusion of cold cadaveric donor renal allografts [3] and cell necrosis that may become prominent at the late stages. Acute tubular necrosis becomes prominent underlying I/R-induced damage, and chronic allograft nephropathy is also related to I/R injury [4]. I/R injury is an acute inflammatory process that may be triggered by hypoxia, which releases cytokines and chemokines in TECs [1, 5].

Reactive oxygen species (ROS) play a critical role in I/R-induced acute kidney injury, as well as progression of fibrosis in various diseases such as hypertension, diabetes, and ureteral obstruction [6]. Renal I/R excessively produces ROS, causes cell damage by lipid peroxidation, DNA breakdown, and protein damage, and leads to AKI [7, 8]. Antioxidant treatment has been used to prevent AKI in various clinical settings and experimental models [9].

2-Methoxyestradiol (2ME2) is an endogenous mammalian metabolite of 17-*β* estradiol with antiproliferative, antitumor, proapoptotic, and antiangiogenic effects [10]. 2ME2 is a well-tolerated small molecule with orally active and potential clinical benefit for treating cancer [11, 12] because 2ME2 causes metaphase arrest that inhibits cell proliferation and induces apoptosis [13]. 2ME2 also downregulates hypoxia inducible factor- (HIF-) 1*α* [14]. In recent studies [15, 16] of focal brain ischemia in rats, 2ME2 treatment inhibited HIF-1*α* expression and protected the part of the brain involved in the antiapoptotic effect of 2ME2. 2ME2 may also inhibit the expression of caspase-3 [15], VEGF, and BNIP3 and, finally, attenuate apoptosis in the brain infarct area that protects the brain from ischemic injury [17]. Furthermore, we previously reported [18] that 2ME2 treatment prolonged survival and reduced LPS-induced kidney injury through HIF-1*α* and NF-*κ*B signaling that significantly reduced IL-1*β* and TNF-*α* levels in septic mice.

Although there are numerous studies on cerebral ischemia in rat models, there are no data available about how 2ME2 affects renal I/R injury. In this study, we investigated the effect of 2ME2 on renal I/R injury. We tested the hypothetical mechanism for the antiapoptotic effect of 2ME2 on mouse renal I/R injury using terminal deoxynucleotidyl transferase mediated dUTP nick end-labeling (TUNEL) staining and the expression of inflammatory cytokines, HIF-1*α*, caspases, and BNIP3. The effect of 2ME2 on ischemia-induced ROS production and cell death in renal cells was also determined.

## 2. Materials and Methods

### 2.1. Animal Model

BALB/c mice were from the National Cheng Kung University Laboratory Animal Center and were cared for according to the guidelines set up by the National Science Council, Taiwan. The experimental protocol adhered to the rules of the Animal Protection Act of Taiwan and was approved by the Institutional Animal Care and Use Committee of Chi-Mei Medical Center. The mice were housed in a temperature- and humidity-controlled (25°C; 60%) room and kept on a 12:12 h light-dark cycle. They were fed standard laboratory chow and water* ad libitum* in the Laboratory Animal Center of Chi-Mei Medical Center. The model of renal I/R injury in the anesthetized mice and the surgical procedures involved were modified as previously described [5].

The mice were divided into the following experimental groups (*n* = 6 in each): (1) sham control, (2) I/R+vehicle, (3) I/R+2ME2 (10 mg/kg), and (4) I/R+2ME2 (20 mg/kg). All the mice were injected (i.p.) either with 2ME2 or with an equivalent volume of vehicle (saline solution) 12 h before and promptly after I/R. After the initial treatment, mice were anesthetized with pentobarbital (30 mg/kg, i.p.), both renal pedicles were isolated using laparotomy and dissection, bilateral ischemia was induced by occluding the renal pedicles with an atraumatic microvascular clamp for 40 min, and then reperfusion was initiated by removing the clamp. After clamps had been released, the incision was closed in two layers with 4-0 sutures. An immediate color change of the kidneys indicating that the blood flow had stopped meant successful occlusion. During reperfusion, the clamps were removed and the blood flow to the kidneys was reestablished and visually verified. After 24 h of reperfusion, the mice were anesthetized, serum samples were collected using heart puncture, the mice were exsanguinated, and both kidneys were removed and stored at −80°C or in formalin for future examination. In another experiment, survival analysis in renal I/R mice with vehicle or 2ME2 (10 or 20 mg/kg, i.p.) treatment (*n* = 6 in each group) was determined for 7 days.

### 2.2. Clinical Blood Chemistry

Mouse serum was separated from whole blood and sent to the Taiwan Mouse Clinic (Taipei, Taiwan), which used standard procedures to measure the levels of serum creatinine and blood urea nitrogen (BUN).

### 2.3. Histological Examination

All kidney tissue specimens were embedded in paraffin. Serial sections (4 *μ*m thick) were processed using hematoxylin and eosin (H&E) stain. Histological examinations were done by an examiner in a blinded fashion. Histological changes due to tubular necrosis or to damage or to the loss of brush border, cast formation, and tubular dilatation were graded as previously described [12]: 0, healthy kidney; 1, minimal necrosis (10% involvement); 2, mild necrosis (10–35% involvement); 3, moderate necrosis (36–75% involvement); and 4, severe necrosis (75% involvement). The cortex, corticomedullary junction (CMJ), and medulla were assessed.

### 2.4. Cell Culture

Rat mesangial cells (RMCs) and rat renal proximal tubular cells (NRK52E) purchased from the American Type Culture Collection (Manassas, USA) were maintained in Dulbecco's modified Eagle's medium supplemented with 10–15% fetal bovine serum and antibiotics and maintained at 37°C in 95% air, 5% CO_2_.

### 2.5. Reverse Transcriptase-Polymerase Chain Reaction (RT-PCR)

Total RNA was extracted using a reagent (RNA-Bee; Tel-Test, Friendswood, TX) and subsequently underwent reverse transcription. HIF-1*α*, BNIP3, caspase-3, IL-1*β*, and TNF-*α* were amplified using RT-PCR with gene-specific primers (supplementary data, Table S1 available online at http://dx.doi.org/10.1155/2014/431524). PCR products were visualized on 2% agarose gels containing ethidium bromide. *β*-Actin was the internal control. The relative quantity of PCR products is expressed as a fold increase relative to controls.

### 2.6. Detecting DNA Fragmentation Using a TUNEL Assay

Frozen sections 10 *μ*m thick were cut using a cryostat (CM1510 S; Leica Microsystems, Taipei, Taiwan) and air-dried. A TUNEL assay was done using a kit (ApoAlert DNA fragmentation assay; Clontech, Palo Alto, CA) according to the manufacturer's instructions. After the assay, a drop of antifade solution was added, the treated portion of the slide was covered with a coverslip, and the edges were sealed with clear nail polish. Slides were viewed within 2 h under a fluorescence microscope (BX 51; Olympus Taiwan, Taipei, Taiwan) equipped with a 40x objective and a dual filter set for green fluorescence (488 nm) and blue fluorescence (461 nm). Images were collected using the microscope fitted with an Olympus America camera and AnalySIS (Soft Imaging Systems, Münster, Germany).

### 2.7. Western Blotting

The kidney tissues and cultured cells were lysed with a buffer containing 1% Triton X-100, 50 mM Tris (pH 7.5), 10 mM EDTA, 0.02% NaN_3_, and a protease inhibitor cocktail (Roche Applied Science, Mannheim, Germany). Following one cycle of freeze-thaw, tissue/cell lysates were centrifuged at 10,000 g for 20 min at 4°C. Lysates were boiled in sample buffer for 5 min. The proteins were then subjected to SDS-PAGE (sodium dodecyl sulfate-polyacrylamide gel electrophoresis) and transferred to a polyvinylidene fluoride (PVDF) membrane (Millipore, Billerica, MA) using a semidry electroblotting system. After they had been blocked with 5% skim milk in PBS, the membranes were incubated with a 1 : 1,000 dilution of primary antibodies—active caspase-9, Bcl-2, Bcl-xL, NF-*κ*B, phosphor-NF-*κ*B (Ser536), HIF-1*α*, and *β*-actin (all from Cell Signaling Technology, Beverly, MA)—at 4°C overnight. The membranes were then washed with 0.05% PBS-Tween 20 and incubated with a 1 : 5,000 dilution of horseradish peroxidase-conjugated anti-rabbit or anti-mouse IgG according to the primary antibodies (Chemicon International, Temecula, CA) at room temperature for 1 h. After they had been washed, the membranes were soaked in ECL solution (PerkinElmer, Boston, MA) for 1 min and then exposed to film (BioMax; Eastman Kodak, Rochester, NY). The relative signal intensity was quantified using ImageJ 1.41 (http://rsbweb.nih.gov/ij/).

### 2.8. DPPH Radical Scavenging Assay

The antioxidant activity of 2ME2 and ascorbic acid (vitamin C) was measured in terms of 1,1-diphenyl-2-picrylhydrazyl (DPPH) (Sigma-Aldrich Inc., St. Louis, MO, USA) free radical scavenging ability. Vitamin C, an antioxidant, was used as a positive control. The highest tested concentration of vitamin C was considered as 100% of scavenging activity. The 2ME2 at different concentrations was placed in a cuvette and 1 mL of DPPH radical in methanol solution (23.7 *μ*g/mL) was added, followed by incubation for 30 min. The decrease in absorbance at 517 nm was determined with a spectrophotometer. All determinations were performed in triplicate. The percent inhibition of DPPH radical by the samples was calculated according to the following formula:

(1) $$\begin{array}{c} \%\;\text{of}\;\text{scavenging}\;\text{of}\;\text{DPPH}=\left[1-\frac{A\left(s\right)}{A\left(c\right)}\right]\times 100, \end{array}$$

where* A*(*c*) is the absorbance of the control (without sample) and* A*(*s*) is the absorbance of the sample at *t* = 30 min.

### 2.9. Intracellular ROS Detection

Intracellular oxidative stress was also measured by dichlorodihydrofluorescein diacetate oxidation. Briefly, 5,000 RMCs were plated in 96-well plates overnight and washed twice with HBSS before experiments. The cells were exposed to 20 *μ*M 5-(and-6)-chloromethyl-2′,7′-dichlorodihydrofluorescein diacetate, acetyl ester (CM-H2DCFDA) (Invitrogen Life Technologies, Carlsbad, CA, USA) for 1 h and then treated with antimycin-A combined with vehicle or 2ME2 at indicated concentrations. H_2_O_2_ (200 *μ*M) was used as the positive control. The fluorescence was read immediately at wavelengths of 485 nm for excitation and 530 nm for emission on a fluorescence plate reader (Fluoroskan Ascent; Thermo Electron Corporation, Milford, MA, USA). The levels of ROS were calculated as percent increases compared with the control, and the control was normalized to 100% of the basal level.

### 2.10. Cell Viability Assay

Cells were seeded at 3 × 10^4^ cells/mL in 24-well dishes and allowed to attach for 8 h and then were exposed to antimycin-A with or without 2ME2 at the indicated concentrations for 24 h. The cells were then incubated with a 1 mg/mL solution of 3-[4,5-dimethylthiazol-2-yl]-2,5-diphenyltetrazolium bromide (MTT) (Sigma-Aldrich, St. Louis, MO, USA) for 3 h. DMSO (dimethyl sulfoxide) (Sigma-Aldrich) was added to the culture, and absorbance was determined at 550 nm. All experiments were done in triplicate.

### 2.11. Statistical Analysis

All values are expressed as the mean ± standard deviation (SD). Statistical analysis was done using SigmaPlot 9.0 (Systat Software, Richmond, CA). The Kruskal-Wallis test and Dunn's test were used. Statistical significance was set at *P* < 0.05. We used Kaplan-Meier analysis to determine the survival distributions and the log-rank test to compare two survival distributions.

## 3. Results

### 3.1. 2ME2 Prolonged Survival and Attenuated Renal Injury in I/R Mice

**Figure 1:**
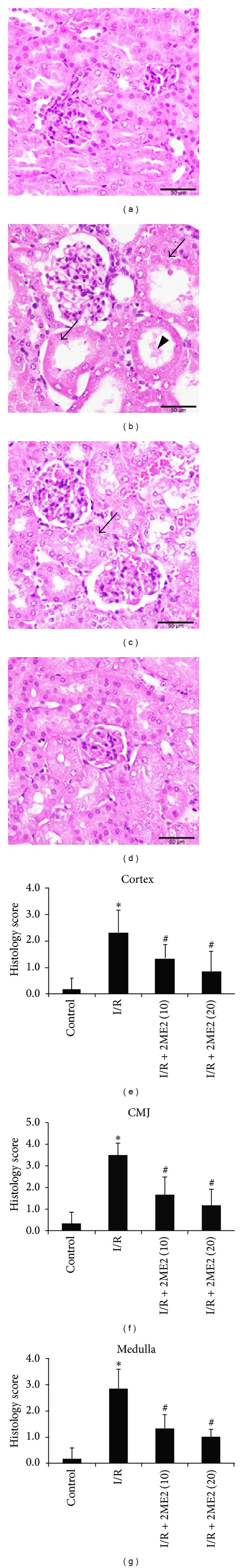
Effect of 2ME2 on renal morphology in renal I/R injury. Renal injury was evidenced by massive TEC necrosis or the damage or loss of brush border (arrows) and by tubular dilation and cast formation (arrowheads). (a) Sham control; (b) I/R-only; (c) I/R + 2ME2: I/R mice injected with 2ME2 (10 mg/kg, i.p.); and (d) I/R+2ME2: I/R mice injected with 2ME2 (20 mg/kg, i.p.). H&E staining was used to evaluate the degree of acute renal tubules damage in renal I/R injury. Representative data are shown. Sections were observed under a microscope (×400). Scale bar is 50 *μ*m. The histological scores of tubular injury in the cortex (e), corticomedullary junction (CMJ) (f), and medulla (g) of all groups of mice are shown. Values are mean ± SD. All groups: *n* = 6. *P < 0.05 compared with control mice and ^#^ *P* < 0.05 compared with I/R-only mice.

**Figure 2:**
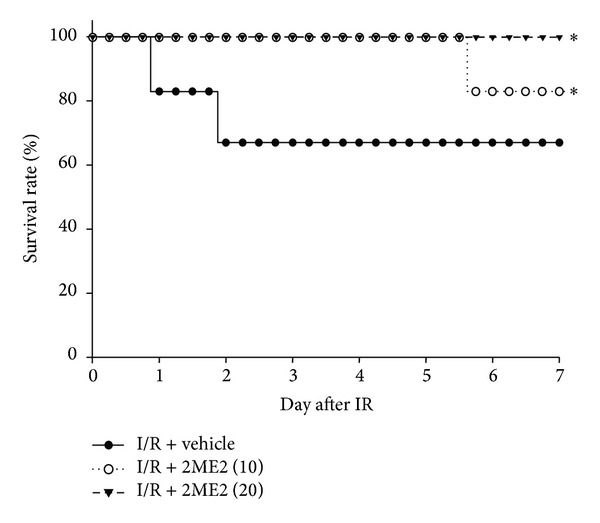
Effects of 2ME2 treatment on survival in mice of renal I/R injury. Mice were injected with 2ME2 (10 or 20 mg/kg, i.p.) or vehicle after renal I/R injury. Survival was determined using Kaplan-Meier analysis and the log-rank test. All groups: *n* = 6. *P < 0.05 versus vehicle group.

To examine the effects of 2ME2 on renal I/R injury, the mice were injected with vehicle or 2ME2 i.p. as described in Materials and Methods. Twenty-four hours after I/R, renal function was assessed based on serum creatinine and BUN levels. The kidney weight and the serum BUN and creatinine concentrations were significantly increasing in I/R-only mice. In I/R+2ME2-treated mice, either in 10 or 20 mg/kg groups, the increase was reduced (Table 1). Histological analysis of the kidney sections showed significant renal tubular injury in I/R group (Figure 1). Kidney I/R-induced tubular cell injuries were characterized by vacuolization, loss of brush borders, sloughing of tubular cells into the lumen, and flattening of the tubular epithelium (Figure 1(b)), which were less frequently seen in the I/R+2ME2 (10 mg/kg) (Figure 1(c)) and I/R+2ME2 (20 mg/kg) groups (Figure 1(d)). The severity of renal I/R injury was also assessed by measuring the histological injury score. The I/R group had higher histological scores than did the sham control group for the cortex, corticomedullary junction (CMJ), and medulla (Figures 1(e), 1(f), and 1(g), resp.). Both the I/R+2ME2 (10 mg/kg) and I/R+2ME2 (20 mg/kg) groups had significantly lower histological scores than did the I/R-only group (*P* < 0.05). Mice survival rates 7 days after there had been renal I/R injury were 67, 83, and 100% for the I/R+vehicle, I/R+2ME2 (10 mg/kg), and I/R+2ME2 (20 mg/kg) groups, respectively (Figure 2).

### 3.2. 2ME2 Attenuated Kidney HIF-1*α*, BNIP3, and Cytokine Expression in I/R Mice

**Figure 3:**
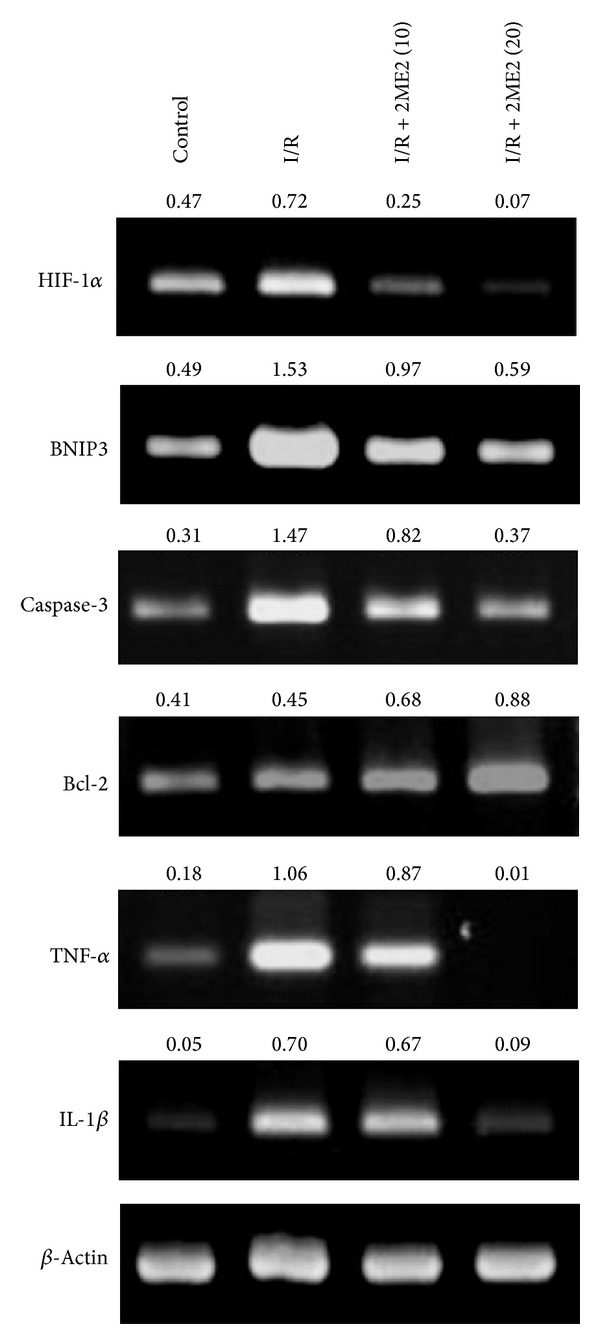
2ME2 reduced HIF-1*α*, BNIP3, caspase-3, Bcl-2, TNF-*α*, and IL-1*β* expression. Gel photographs depicting mRNA expression of HIF-1*α*, BNIP3, caspase-3, Bcl-2, and the cytokines TNF-*α* and IL-1*β*, using RT-PCR in renal tissue extracts from mice treated with saline or I/R-only or I/R+2ME2 (10 mg/kg) or I/R+2ME2 (20 mg/kg). *β*-Actin was the internal control. The ratios of HIF-1*α*, BNIP3, caspase-3, Bcl-2, TNF-*α*, and IL-1*β* to *β*-actin are shown (numbers above the top three panels), respectively. Data are representative of three independent experiments.

We investigated the effects of 2ME2 on HIF-1*α* and its downstream genes BNIP3 and cytokines in kidney of I/R mice. RT-PCR data showed that kidney HIF-1*α* expression was induced by I/R but substantially lower in 2ME2-treated mice (Figure 3). Similarly, mRNA expression levels of the proapoptotic genes, BNIP3 and caspase-3, and the cytokines IL-1*β* and TNF-*α* (Figure 3) were apparently higher in I/R-only mice than in I/R+2ME2-treated (10 mg/kg) mice.

### 3.3. Effect of 2ME2 on Apoptosis in Renal I/R Injury

**Figure 4:**
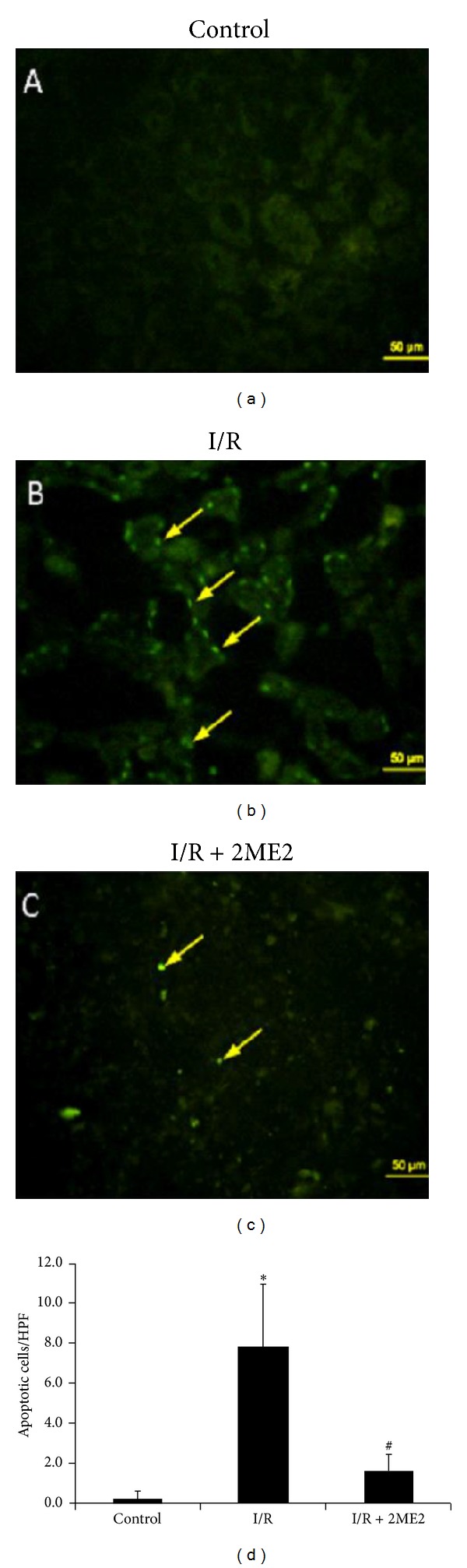
TUNEL staining of kidneys. Apoptotic cells in the kidneys of each group of mice were determined using TUNEL staining. All groups: *n* = 6. (a) There is no fluorescent intensity in the control kidney with saline treatment. (b) Apoptotic cells displaying high fluorescence intensity were detected in sections from I/R-only kidneys; however, (c) the green fluorescence of apoptotic nuclei is rare in tubular epithelial cells of the kidneys with 2ME2 (20 mg/kg) treatment. Sections were observed under a fluorescence microscope (×400). Scale bar is 50 *μ*m. Representative data are shown. (d) Apoptotic cell counts in kidneys are averages of 10 high power fields (HPFs) from each group of mice. All groups: *n* = 6. *P < 0.05 compared with control mice and ^#^ *P* < 0.05 compared with I/R-only mice.

We next determined whether 2ME2 reduces apoptosis in renal I/R injury. Representative kidney histological sections from sham control, I/R-only, and I/R+2ME2-treated mice were stained using* TUNEL* assay to detect apoptotic cells. Apoptosis was more prominent in the kidney sections from I/R-only mice than in those from sham control mice (Figure 4). A large number of apoptotic nuclei in renal tubular epithelial cells were detected in I/R-only kidneys (Figure 4(b)). Compared with I/R-only mice, markedly fewer apoptotic nuclei in kidney were detected in I/R+2ME2-treated mice (Figures 4(c) and 4(d)).

### 3.4. 2ME2 Reduced Renal I/R-Induced HIF-1*α*, Activated Caspase-9, and pNF-*κ*B but Induced BCL-2 and BCL-xL Protein in Mice

**Figure 5:**
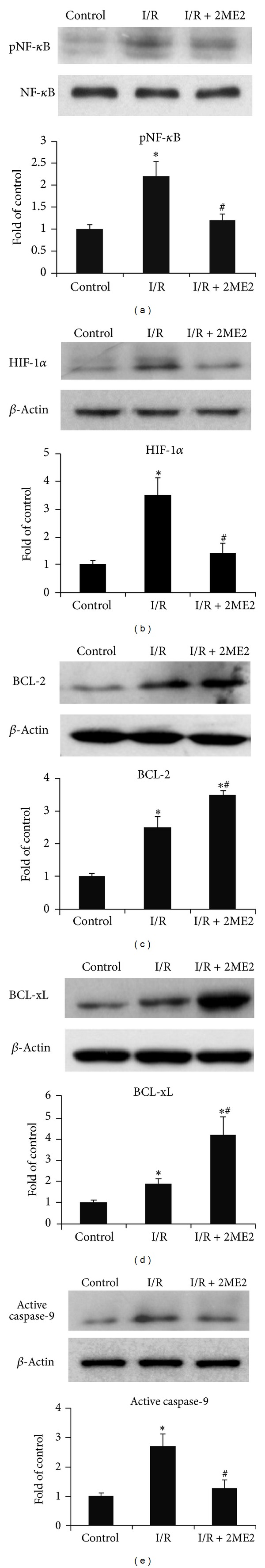
2ME2 reduced expression of HIF-1*α*, activated caspase-9, and pNF-*κ*B but increased antiapoptotic BCL-xL and BCL-2. Expression levels of HIF-1*α* (a), pNF-*κ*B (b), BCL-2 (c), BCL-xL (d), and activated caspase-9 (e) in kidneys were determined using western blotting in three groups: sham control: mice undergoing the same procedure without occlusion of the renal pedicle; I/R-only: mice given renal I/R challenge combined with vehicle (normal saline) treatment; I/R+2ME2: I/R-only mice injected with 2ME2 (10 mg/kg, i.p.). *β*-Actin was the internal control. Representative data are shown. All groups: *n* = 6. *P < 0.05 compared with control mice and ^#^ *P* < 0.05 compared with I/R-only mice.

Western immunoblots of kidney lysates were used to confirm whether 2ME2 protects against apoptosis by inhibiting HIF-1*α* protein expression. Under baseline conditions, caspase-3 activation was not detectable in the kidneys of any of the mice. Protein levels of HIF-1*α* (Figure 5(a)), pNF-*κ*B (Figure 5(b)), and activated caspase-9 (Figure 5(e)) were higher in the I/R-only group than in the sham control and I/R+2ME2 group. However, BCL-2 (Figure 5(c)) and BCL-xL (Figure 5(d)) expression were higher in the I/R+2ME2 group than in the I/R-only group.

### 3.5. 2ME2 Attenuated ROS Production and Increased Cell Viability in Ischemic Renal Cells* In Vitro*

**Figure 6:**
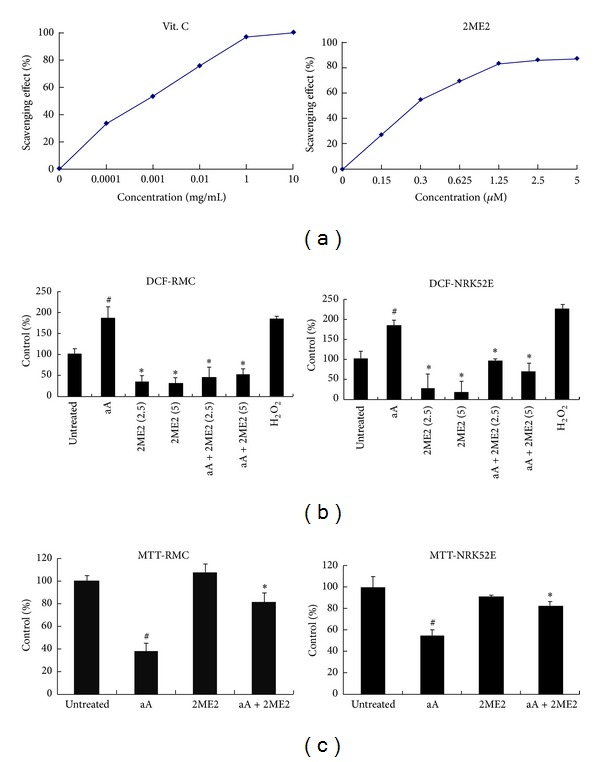
2ME2 reduced ROS production and increased cell survival in antimycin-A (Aa)-treated renal cells. (a) Direct free radical scavenging activity of 2ME2 determined by the DPPH radical scavenging assay. Values represent the data of three independent experiments. Vitamin C was used as a positive control compound. (b) Cells were treated with antimycin-A 10 *μ*M with saline or 2ME2 (2.5 or 5 *μ*M) for 1 h. Quantitation of ROS generation in RMCs and NRK52E cells using CM-H2DCFDA assay. (c) RMCs and NRK52E cells were treated with antimycin-A 10 *μ*M with saline or 2ME2 2.5 *μ*M for 16 h. Cell survival was determined using MTT assay. All groups: *n* = 3. ^#^ *P* < 0.05 compared to untreated group and *P < 0.05 compared to antimycin-A group.

ROS play a critical role in I/R-induced renal injury; however, the effect of 2ME2 on ROS production has not been reported. To determine whether oxidative stress is involved in 2ME2's renal protective mechanism under I/R condition, we evaluated the direct ROS scavenging effect of 2ME2 in antimycin-A-treated RMCs and NRK52E. Antimycin-A is an inhibitor of oxidative phosphorylation used for chemical hypoxia. As shown in the DPPH radical scavenging assay (Figure 6(a)), 2ME2 scavenged the free radicals in a concentration-dependent manner similar to the antioxidant vitamin C. The antioxidative ability of 2EM2 was further measured by CM-H2DCFDA staining. ROS was noted to increase in RMCs and NRK52E under antimycin-A exposure compared with untreated control, but its levels were markedly inhibited by 2ME2 (Figure 6(b)). Moreover, we treated RMCs and NRK52E cells with antimycin-A 10 *μ*M combined with saline or 2ME2 2.5 *μ*M for 16 h and determined the cell viability using MTT assay. 2ME2 significantly reduced cell death in antimycin-A-treated cells (Figure 6(c)).

## 4. Discussion

The mechanisms of kidney injury exist within a large network of signaling pathways driven by interplay of inflammatory cytokines, ROS, and apoptotic factors [7]. We evaluated the therapeutic effects of 2ME2 on renal I/R injury in a murine model. We found that 2ME2 reduced renal I/R-induced renal dysfunction through decreasing pNF-*κ*B, TNF-*α*, IL-1*β*, BNIP3, activated caspase-9, and caspase-3 but increasing the expression of antiapoptotic BCL-2 and BCL-xL in renal tissue. Moreover, in ischemic renal cells, 2ME2 reduced ROS production and increased cell viability. These results implied that the inhibition of ROS and proinflammatory cytokines by 2ME2 protected renal function and controlled tubular damage in kidneys undergoing I/R injury.

I/R injury is considered an inflammatory process in which TECs acquire a proinflammatory phenotype and start to release cytokines and chemokines upon hypoxic injury [2]. Renal injury is amplified after reperfusion such that there is, in the damaged areas, a massive influx of neutrophils that induce adhesion molecules and release proteases [1, 5]. In these conditions, TECs undergo necrosis, and necrotic products activate Toll-like receptor (TLR) signaling pathways, which amplifies the inflammatory response. During hepatic I/R, NF-*κ*B activation through TLR-4 binding promotes hepatic injury [1]. NF-*κ*B is a transcription factor consisting of Rel family proteins known to promote the transcription of key immunomodulatory and proinflammatory genes, such as TNF-*α* and IL-1*β* [18]. TNF-*α* and IL-1*β* upregulated and released from resident kidney macrophages and renal parenchymal cells promote organ damage by inducing apoptosis and by recruiting neutrophils during renal I/R [5]. We recently reported [18] that 2ME2 attenuated the activation of inducible NF-*κ*B, which affects the level of TNF-*α* and IL-1*β* in LPS-challenged macrophages. In this renal I/R experimental model, we found that 2ME2 significantly reduced the protein level of pNF-*κ*B and showed a trend toward decreasing TNF-*α* and IL-1*β* mRNA production in the I/R+2ME2-treated groups, while it was increased in the I/R-only group, which indicates 2ME2's renoprotective effect against hypoxic stress. These results support that the protective effects of 2ME2 in renal I/R injury are provided by the decreases in proinflammatory cytokines.

Regulating apoptosis and inflammation is also involved in activating the transcription factor HIF-1*α* [16]. Neuron-specific knockouts of HIF-1*α* to hypoxic-ischemic damage in a global mouse ischemia model suggested that decreasing the level and loss of the proapoptotic function of HIF-1*α* could be neuroprotective [19]. In kidney, hypoxia triggers the accumulation of HIF-1*α* in tubular cells [20]. However, the accumulation of HIF-1*α* may process renoprotective potentials for increasing oxygen availability [21, 22] and promoting cellular adaptive mechanisms under different conditions [23]. Although HIF activation leads to the expression of a variety of adaptive genes in a coordinated manner that may provide a renoprotective effect, in brain tissue, HIF-1*α* is an essential proapoptotic regulator [17]. In this study, HIF-1*α* expression was concomitant with proinflammatory cytokines and 2ME2 treatment inhibited HIF-1*α* expression and improved renal function and survival in I/R injury. The beneficial or detrimental role of HIF-1*α* in renal I/R injury remains to be further investigated.

HIF-1*α* protein levels that peaked during the initial reperfusion phase and remained elevated up to 24 h after hypoxic-ischemic brain injury have been detected [24]. 2ME2 is known to protect rats from ischemic brain injury by reducing the cortical expression of HIF-1*α* and of its downstream apoptotic genes, BNIP3 and caspase-3 [17]. HIF-1*α* is expressed and colocalized with BNIP3 and TUNEL-positive cells [17]. Furthermore, BNIP3 is a dimeric mitochondrial proapoptotic protein that influences mitochondrial function in the early apoptotic process and can counteract Bcl-2-induced inhibition of apoptosis or cytochrome C release from mitochondria into the cytoplasm [25]. In the present study, we found that 2ME2 treatment decreased HIF-1*α* and BNIP3 expression, which indicates the possibility of using 2ME2 to downregulate the apoptotic pathway in injured renal tissue. In addition, expression of the apoptosis-related genes p53 (a tumor suppressor), caspase-9, and caspase-3 increased in a time course similar to that of the increase in HIF-1*α* production after brain ischemic injury [26]. In this global ischemia-hypotension rat model, HIF-1*α* led to apoptosis by binding with p53 in nuclei, which activates caspase-9 and caspase-3 in the cytosol by releasing cytochrome C. Hence, 2ME2 may theoretically inhibit proapoptotic pathways by reducing the HIF-1*α* level in ischemic tissue. Our present data showed that renal cell apoptosis and the expression of activated caspase-9 and caspase-3 were elevated after renal I/R injury. In contrast, 2ME2 treatment attenuated the expression of both caspases associated with the actions of HIF-1*α*. This implied that the therapeutic effect of 2ME2 on the decrease of TUNEL-positive cells and the preservation of renal tissue could also act by inhibiting HIF-1*α*-induced caspase-3 expression, as previously reported [27]. The renoprotection imparted by 2ME2 in this study is probably via multiple pathways after renal I/R. We also found that the expression of antiapoptotic Bcl-2 and Bcl-xL increased after I/R and that the production of Bcl-2 was promoted with 2ME2 treatment. Elevation of Bcl-2 and Bcl-xL by 2ME2 may also inhibit apoptosis [26] and provide cell protection during I/R injury. However, ischemia induces HIF-1*α* expression both early and late and causes both apoptosis and cell survival, respectively [16].

ROS is another important mediator in I/R-induced acute kidney injury. Renal I/R excessively produces ROS, causes lipid peroxidation, DNA, and protein damage, and leads to renal cell injury [7, 8]. In this study, we found that 2ME2 can scavenge ROS and reduce chemical hypoxia induced cell death. As antioxidant treatment has been used to prevent AKI in various clinical settings and experimental models [9], we considered that 2ME2 may play a role as an antioxidant and anti-inflammatory agent and could have therapeutic potential in renal I/R injury.

In conclusion, 2ME2 treatment improves renal functional and mice survival in renal I/R injury. The renoprotective effects of 2ME2 on renal I/R damage in mice were mediated, at least in part, by early ROS and cytokine inhibition, which constrained apoptosis and inflammation. We suggest that 2ME2 might have the potential to prevent acute renal injury during the clinical events, such as renal transplantation and major cardiovascular surgery.

## Supplementary Material

Table S1: Primer pairs used in this study.

## Figures and Tables

**Table 1:** Kidney weight and serum BUN and creatinine levels in renal ischemia/reperfusion (I/R) mice combined with or without 2ME2 treatment.

	Sham control	I/R-only	I/R + 2ME2 (10 mg/kg, i.p.)	I/R + 2ME2 (20 mg/kg, i.p.)
Kidney weight (%)	1.04 ± 0.35	2.16 ± 1.65∗	1.38 ± 0.67^#^	1.15 ± 0.43^#^
BUN (mg/dL)	18.80 ± 1.90	52.70 ± 12.50∗	28.70 ± 1.70^#^	21.40 ± 3.50^#^
Creatinine (*μ*g/dL)	0.14 ± 0.03	1.66 ± 0.14∗	0.28 ± 0.06^#^	0.19 ± 0.04^#^

Sham control: mice undergoing the same procedure without occlusion of the renal pedicle; I/R-only: mice given renal I/R challenge combined with vehicle (normal saline) treatment; I/R + 2ME2: mice given I/R combined with 2ME2 (10 or 20 mg/kg, i.p.) treatment; and i.p.: intraperitoneally. Kidney weight (%): percentage of body weight of each kidney. Values are mean ± SD. All groups: *n* = 6. ∗*P* < 0.05 compared with sham control mice; ^#^ *P* < 0.05 compared with I/R-only mice.

## References

[1] Sadis C, Teske G, Stokman G (2007). Nicotine protects kidney from renal ischemia/reperfusion injury through the cholinergic anti-inflammatory pathway. *PLoS ONE*.

[2] Munshi R, Hsu C, Himmelfarb J (2011). Advances in understanding ischemic acute kidney injury. *BMC Medicine*.

[3] Burns AT, Davies DR, Mclaren AJ, Cerundolo L, Morris PJ, Fuggle SV (1998). Apoptosis in ischemia/reperfusion injury of human renal allografts. *Transplantation*.

[4] Hayashi T, Saitou Y, Nose K, Nishioka T, Ishii T, Uemura H (2008). Efficacy of carvedilol for ischemia/reperfusion-induced oxidative renal injury in rats. *Transplantation Proceedings*.

[5] Kher A, Meldrum KK, Hile KL (2005). Aprotinin improves kidney function and decreases tubular cell apoptosis and proapoptotic signaling after renal ischemia-reperfusion. *Journal of Thoracic and Cardiovascular Surgery*.

[6] Kim J, Seok YM, Jung K, Park KM (2009). Reactive oxygen species/oxidative stress contributes to progression of kidney fibrosis following transient ischemic injury in mice. *The American Journal of Physiology: Renal Physiology*.

[7] Sabbahy ME, Vaidya VS (2011). Ischemic kidney injury and mechanisms of tissue repair. *Wiley Interdisciplinary Reviews: Systems Biology and Medicine*.

[8] Johnson KJ, Weinberg JM (1993). Postischemic renal injury due to oxygen radicals. *Current Opinion in Nephrology and Hypertension*.

[9] Dobashi K, Ghosh B, Orak JK, Singh I, Singh AK (2000). Kidney ischemia-reperfusion: modulation of antioxidant defenses. *Molecular and Cellular Biochemistry*.

[10] Ricker JL, Chen Z, Yang XP, Pribluda VS, Swartz GM, van Waes C (2004). 2-Methoxyestradiol inhibits hypoxia-inducible factor 1*α*, tumor growth, and angiogenesis and augments paclitaxel efficacy in head and neck squamous cell carcinoma. *Clinical Cancer Research*.

[11] Pribluda VS, Gubish ERJ, LaVallee TM, Treston A, Swartz GM, Green SJ (2000). 2-Methoxyestradiol: an endogenous antiangiogenic and antiproliferative drug candidate. *Cancer and Metastasis Reviews*.

[12] Tevaarwerk AJ, Holen KD, Alberti DB (2009). Phase i trial of 2-methoxyestradioI NanoCrystal dispersion in advanced solid malignancies. *Clinical Cancer Research*.

[13] Chua YS, Chua YL, Hagen T (2010). Structure activity analysis of 2-methoxyestradiol analogues reveals targeting of microtubules as the major mechanism of antiproliferative and proapoptotic activity. *Molecular Cancer Therapeutics*.

[14] Mabjeesh NJ, Escuin D, LaVallee TM (2003). 2ME2 inhibits tumor growth and angiogenesis by disrupting microtubules and dysregulating HIF. *Cancer Cell*.

[15] Li Y, Xia Z, Chen L (2011). HIF-1-alpha and survivin involved in the antiapoptotic effect of 2ME2 after global ischemia in rats. *Neurological Research*.

[16] Yeh S, Ou L, Gean P, Hung J, Chang W (2011). Selective inhibition of early-but not late-expressed HIF-1α is neuroprotective in rats after focal ischemic brain damage. *Brain Pathology*.

[17] Chen C, Hu Q, Yan J (2007). Multiple effects of 2ME2 and D609 on the cortical expression of HIF-1α and apoptotic genes in a middle cerebral artery occlusion-induced focal ischemia rat model. *Journal of Neurochemistry*.

[18] Yeh C-H, Chou W, Chu C-C (2011). Anticancer agent 2-methoxyestradiol improves survival in septic mice by reducing the production of cytokines and nitric oxide. *Shock*.

[19] Helton R, Cui J, Scheel JR (2005). Brain-specific knock-out of hypoxia-inducible factor-1α reduces rather than increases hypoxic-ischemic damage. *Journal of Neuroscience*.

[20] Rosenberger C, Mandriota S, Jürgensen JS (2002). Expression of hypoxia-inducible factor-1α and -2α in hypoxic and ischemic rat kidneys. *Journal of the American Society of Nephrology*.

[21] Lee J, Bae S, Jeong J, Kim S, Kim K (2004). Hypoxia-inducible factor (HIF-1)*α*: Its protein stability and biological functions. *Experimental and Molecular Medicine*.

[22] Matsumoto M, Makino Y, Tanaka T (2003). Induction of renoprotective gene expression by cobalt ameliorates ischemic injury of the kidney in rats. *Journal of the American Society of Nephrology*.

[23] Bernhardt WM, Warnecke C, Willam C, Tanaka T, Wiesener MS, Eckardt K (2007). Organ protection by hypoxia and hypoxia-inducible factors. *Methods in Enzymology*.

[24] Calvert JW, Cahill J, Yamaguchi-Okada M, Zhang JH (2006). Oxygen treatment after experimental hypoxia-ischemia in neonatal rats alters the expression of HIF-1*α* and its downstream target genes. *Journal of Applied Physiology*.

[25] Yeh C-H, Cho W, So EC (2011). Propofol inhibits lipopolysaccharide-induced lung epithelial cell injury by reducing hypoxia-inducible factor-1α expression. *British Journal of Anaesthesia*.

[26] Li Y, Zhou C, Calvert JW, Colohan ART, Zhang JH (2005). Multiple effects of hyperbaric oxygen on the expression of HIF-1α and apoptotic genes in a global ischemia-hypotension rat model. *Experimental Neurology*.

[27] Chen W, Jadhav V, Tang J, Zhang JH (2008). HIF-1*α* inhibition ameliorates neonatal brain injury in a rat pup hypoxic-ischemic model. *Neurobiology of Disease*.

