# Supplementary material for: Anticancer Drug 2-Methoxyestradiol Protects against Renal Ischemia/Reperfusion Injury by Reducing Inflammatory Cytokines Expression
Source: Biomed Res Int. 2014 Aug 6;2014:431524. doi: 10.1155/2014/431524 (PMC4151070; doi:10.1155/2014/431524)
Supplement: Supplementary file 1 — Table S1: Primer pairs used in this study. [file 431524.f1.pdf]

Supplementary data

Material and Methods

Table S1. Primer pairs used in this study.

**Factor**	**Primer sequence (5'-3')**
HIF-1α	F-GCACTAGACAAAGTTCACCTGAGA
	R-CGCTATCCACATCAAAGCAA
BNIP3	F-GAACTGCACTTCAGCAATGG
	R-GCCAGCAGATGAGACAGTAA
Casapase-3	F-GCGGGGAGCTTGGAACGCTA
	R-ACCCCGGCAGGCCTGAATGA
Bcl-2	F-AGCAACCCAATGCCCGCTGT
	R-TGTGGCCCAGGTATGCACCCA
IL-1β	F-GTGGCTGTGGAGAAGCTGTGGC
	R-TGGGTCCGACAGCACGAGGC
TNF-α	F-GAGTGACAAGCCTGTGCCCA
	R-CCCTTCTCCAGCTGGAAGA
β-actin	F-GGGAATGGGTCAGAAGGACT
	R-TTTGATGTCACGCACGATTT
